# (E)-N′-(1-(7-Hydroxy-2-Oxo-2H-Chromen-3-Yl) Ethylidene) Benzohydrazide, a Novel Synthesized Coumarin, Ameliorates Isoproterenol-Induced Myocardial Infarction in Rats through Attenuating Oxidative Stress, Inflammation, and Apoptosis

**DOI:** 10.1155/2020/2432918

**Published:** 2020-03-06

**Authors:** Anouar Feriani, Emna Khdhiri, Meriam Tir, Afoua Elmufti, Nizar Tlili, Raouf Hajji, Houcine Ammar, Noureddine Allouche, Souhir Abid, Lakhdar Ghazouani, Kais Mnafgui

**Affiliations:** ^1^Research Unit of Macromolecular Biochemistry and Genetics, Faculty of Sciences of Gafsa, 2112 Gafsa, Tunisia; ^2^Laboratoire de Chimie Appliquée “Hétérocycles Corps Gras & Polymères”, Faculté des Sciences, Université de Sfax, 3038 Sfax, Tunisia; ^3^Laboratoire d'Ecologie, de Biologie et de Physiologie des Organismes Aquatiques, LR18ES41, Faculté des Sciences de Tunis, Université Tunis EL Manar, 2092 Tunis, Tunisia; ^4^Institut Supérieur des Sciences et Technologies de l'Environnement, Université de Carthage, Tunisia; ^5^Département de Biologie (UR 13/ES25), Faculté des Sciences de Tunis, Université Tunis El-Manar, Tunis 2092, Tunisia; ^6^Internal Medicine Department, Sidi Bouzid Hospital, Ibn Eljazzar Faculty of Medicine, University of Sousse, Sousse, Tunisia; ^7^Laboratory of Organic Chemistry LR17ES08 (Natural Substances Team), Faculty of Sciences of Sfax, University of Sfax, Tunisia; ^8^Laboratory of Animal Physiology, Faculty of Sciences of Sfax, University of Sfax, P.O. Box 95, Sfax 3052, Tunisia

## Abstract

The present study was directed to investigate the effect of precotreatment with (E)-N′-(1-(7-hydroxy-2-oxo-2H-chromen-3-yl) ethylidene) benzohydrazide (7-hyd.HC), a novel potent synthesized coumarin, on isoproterenol- (ISO-) induced myocardial infarction (MI) in rats. The hydrazone compound was characterized by IR, 1D, and 2D NMR analyses. Experimental induction of MI in rats was established by ISO (85 mg/kg/day, s.c) for two consecutive days (6th and 7th days). 7-hyd.HC or sintrom was given for 7 days prior and simultaneous to ISO injection. 7-hyd.HC offered a cardiopreventive effect by preventing heart injury marker leakage (LDH, ALT, AST, CK-MB, and cTn-I) from cardiomyocytes and normalizing cardiac function and ECG pattern, as well as improving lipid profile (TC, TG, LDL-C, and HDL-C), which were altered by ISO administration. Moreover, 7-hyd.HC precotreatment significantly mitigated the oxidative stress biomarkers, as evidenced by the decrease of lipid peroxidation and the increased level of the myocardial GSH level together with the SOD, GSH-Px, and catalase activities. 7-hyd.HC inhibited the cardiac apoptosis by upregulating the expression of Bcl-2 and downregulating the expression of Bax and caspase-3 genes. In addition, 7-hyd.HC reduced the elevated fibrinogen rate and better prevented the myocardial necrosis and improved the interstitial edema and neutrophil infiltration than sintrom. Overall, 7-hyd.HC ameliorated the severity of ISO-induced myocardial infarction through improving the oxidative status, attenuating apoptosis, and reducing fibrinogen production. The 7-hyd.HC actions could be mediated by its antioxidant, antiapoptotic, and anti-inflammatory capacities.

## 1. Introduction

Cardiovascular disease (CVD) is one of the global health emergencies of the 21st century, which caused 17.9 million dead people in 2015 and this may rise up to 23.6 million by the year 2030 [1]. Myocardial infarction (MI) is the commonest form of this disease which is characterized by insufficient supply of myocardial oxygen compared to demand leading to myocardial hypoxia and necrosis [2]. More insights into the mechanism of myocardial infarction through various researches have revealed the role of oxidative stress in promoting lipid peroxidation, inflammation, and apoptosis in the myocardium by excessive production of free radicals [3, 4].

Recognizing the gravity of CVD will require genuine consideration, particularly for MI [5]. Clinical and experimental searches have reported that synthetic drug therapy such as statins can mitigate or prevent myocardial damage and heart failure through inhibition of inflammation and enhancement of endothelial function [6, 7, 8]. However, it has been revealed that statin treatment could reduce the endogenous antioxidants resulting in the decreases of the body's resistance to oxidative stress [9]. Hence, many researchers have focused for the identification of new therapeutic approaches to treat myocardial infarction with minimal side effects [10]. Recent reports have demonstrated that plant-based phytoconstituents has been used for the counteracting effect and treatment of cardiac dysfunction [11]. Nowadays, several attempts have focused on coumarins, as an abundant secondary metabolite, considered an effective bioactive molecule that present a promising therapeutic option. Coumarins are found in several plant families and essential oils and are used as fragrant additives in food and cosmetics [12]. Many reports have suggested that the coumarins possess a huge array of biological roles, such as antithrombotic, neuroprotective, antidiabetic, anticoagulant, anti-inflammatory, antioxidant, and antiplasmodial activities [13–19]. Due to technical advancement, the coumarins and its derivatives are becoming a potential source for new drug discovery [20].

Thus, due to the side effects of the synthetic drugs from one hand and the beneficial role of coumarins and derivatives from the other hand, the present study was designed to investigate, for the first time, the potential preventive capacity of a novel derivative of coumarin, (E)-N′-(1-(7-hydroxy-2-oxo-2H-chromen-3-yl) ethylidene) benzohydrazide, against isoproterenol-induced MI in male Wistar rats. The synthesized compound (7-hyd.HC) was checked for quality by 1H and 13C NMR spectroscopy, and IR. In order to explore the mechanism of the cardioprotection of the newly synthesized hydrazone coumarin, the ECG pattern, the heart rate, the plasmatic cardiac biomarkers, the status of pro- and antioxidants, myocardial proapoptotic and antiapoptotic factors, measurement of myocardial infarction size, and cardiac histopathology were elucidated by comparison to an acenocoumarol (sintrom) drug.

## 2. Materials and Methods

### 2.1. Drugs and Chemicals

Benzohydrazide, 2,4-dihydroxybenzaldehyde, silica gel, piperidine, methyl 3-oxobutanoate, acetic acid, ethyl acetate, hexane, ethanol, and isoproterenol hydrochloride powder were obtained from Sigma-Aldrich, St. Louis, USA. Sintrom (4 mg tablets) was obtained from the laboratory of Novartis Pharma, Tunisia. The remaining chemicals used were of analytical grade.

### 2.2. Apparatus for Chemistry Analysis

The melting point (mp) was determined with Kofler bench. The TF-IR spectrum was recorded on a Perkin Elmer spectrum 100 FTIR Spectrometer. ^1^H NMR (400 MHz) and ^13^C NMR (100 MHz) spectra were recorded with a Bruker 400 MHz spectrometer. Chemical shifts (*δ*) are expressed in parts per million (ppm) using TMS as an internal standard. Spin multiplicities are given as s (singlet), d (doublet), and t (triplet).

### 2.3. Synthesis of (E)-N′-(1-(7-Hydroxy-2-Oxo-2H-Chromen-3-Yl) Ethylidene) Benzohydrazide (7-hyd.HC)

**Scheme 1:**
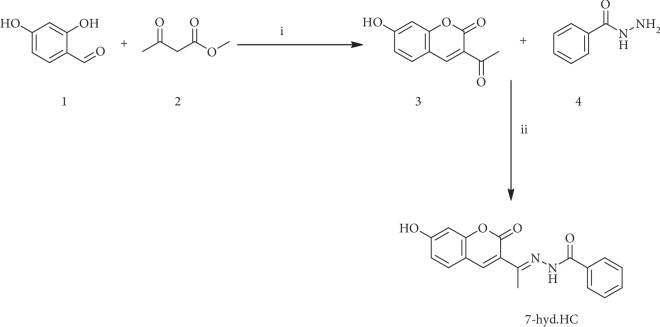
Synthesis of compound 7-hyd.HC: i: ethanol, 80°C, piperidine; ii: ethanol, 80°C, acetic acid.

The synthetic strategy adopted to obtain the target compound was depicted in Scheme 1. The starting reactive, 3-acetyl-7-hydroxy-2H-chromen-2-one (3), was prepared by 2,4-dihydroxybenzaldehyde (1) (3.6 mmol), ethyl acetoacetate (2) (3.6 mmol), and a catalytic amount of piperidine in 15 ml ethanol by Knoevenagel reaction. The condensation of the 3-acetyl coumarin (3) (1 eq) with benzohydrazide (4) (1 eq) in 15 mL ethanol using acetic acid as a catalyst under reflux condition for 8 h afforded a solid isolated by filtration. The progress of the reaction in all cases was monitored by thin-layer chromatography (TLC) examination using ethyl acetate and hexane (1 : 4 *v*/*v*). The resulting crude product was purified by passing through a column of silica gel (60-120 mesh) with ethyl acetate and hexane (1 : 4 *v*/*v*) as eluent and dried under vacuum at 50°C to obtain target molecule (E)-N′-(1-(7-hydroxy-2-oxo-2H-chromen-3-yl) ethylidene) benzohydrazide (7-hyd.HC) in 46% yield as pale white solid: mp > 264°C.

### 2.4. Mechanism

**Scheme 2:**
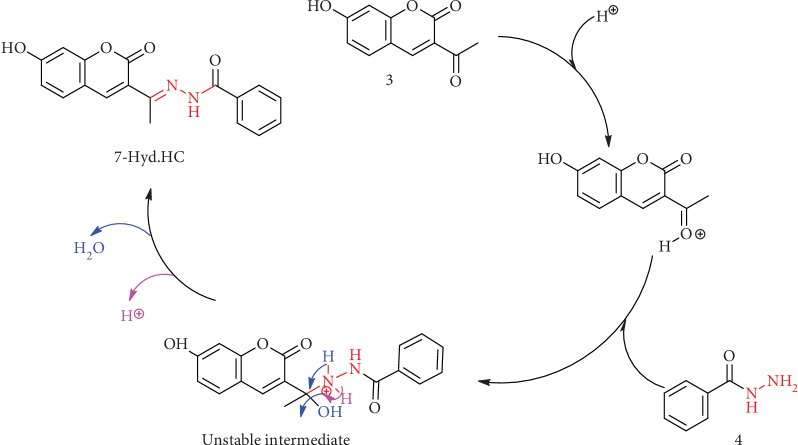
Mechanism of the formation of 7-hyd.HC

As shown in Scheme 2, the addition of benzohydrazide (4) through the free electron pair of the NH_2_ group after the protonation of the ketonic oxygen of 3-acetyl coumarin (3) will result in the formation unstable intermediate. The second step of this mechanism results in deprotonation, and elimination of a molecule of water gives the new 7-hyd.HC.

### 2.5. Characterization of Compound and Their Conformational Studies

**Figure 1:**
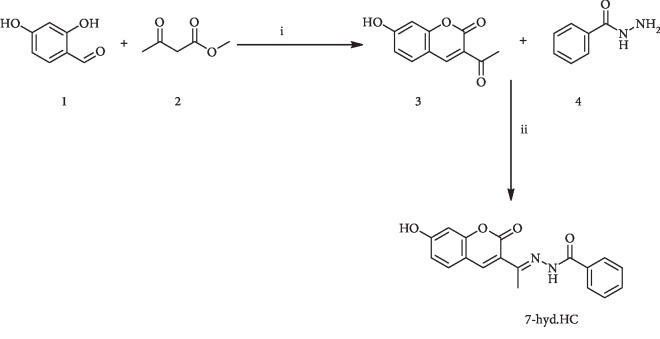
FT-IR spectrum of compound 7-hyd.HC.

**Figure 2:**
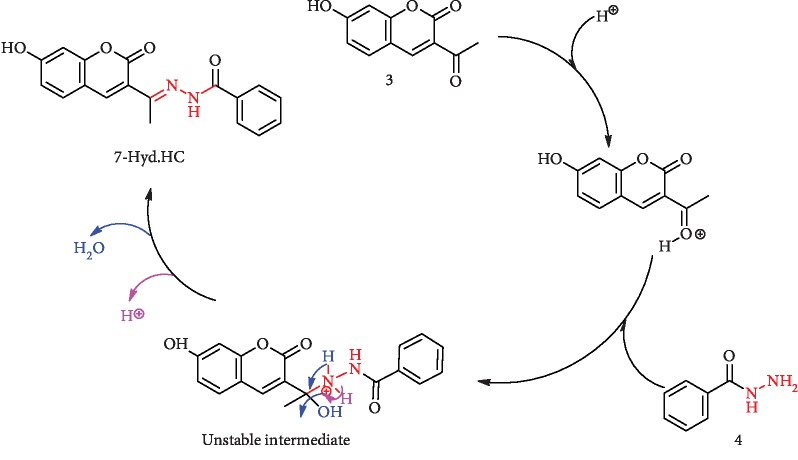
^1^HNMR spectrum (DMDO-d_6_, 400 MHz) of compound7-hyd.HC.

**Figure 3:**
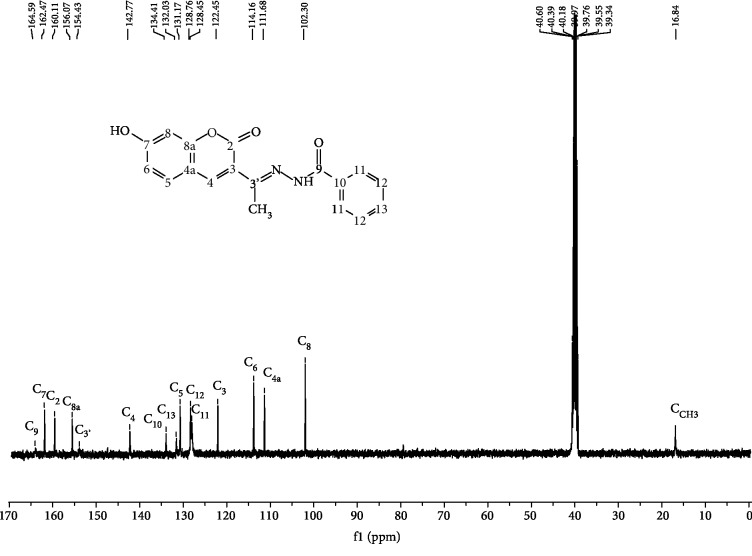
^13^C NMR spectrum (DMDO-d_6_, 100 MHz) of compound 7-hyd.HC.

**Figure 4:**
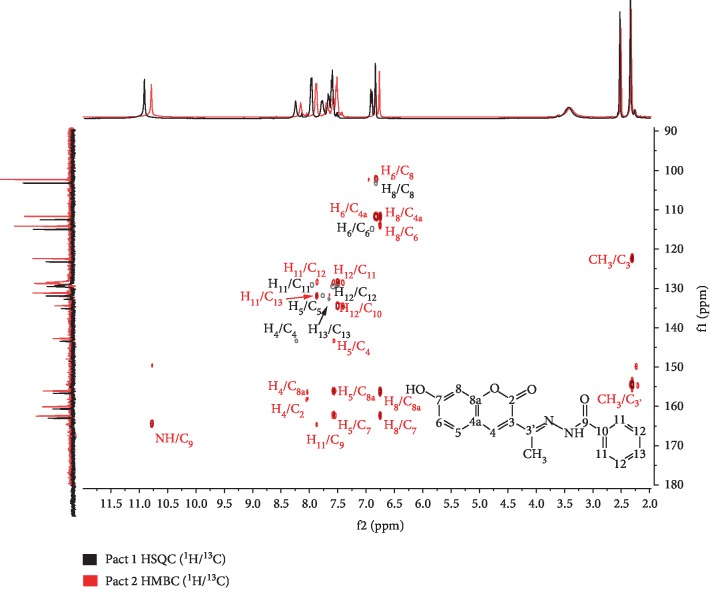
Correlations *J*_C−H_ from HSQC (in black) and HMBC (in red) spectra of compound 7-hyd.HC.

The synthesized compound was confirmed by IR, 1D, and 2D NMR. The IR spectrum of the new compound Figure 1 displayed N-H amide (3305 cm^−1^), hydroxyl group (3225 cm^−1^), lactone (1726 cm^−1^), carbonyl hydrazone (1706 cm^−1^), and imine (1620 cm^−1^) absorptions. The NMR data of the compound 7-hyd.HC is summarized in Table 1. In ^1^H NMR Figure 2, the appearance of one singlet for only one NH group and only one OH proton in the low-field region at 10.87 ppm confirmed the structure of the new compound. Morever, in the ^13^C NMR Figure 3, the signals were observed at the regions *δ*C 154.4, 160.1, and 164.5 ppm due to the presence of C=N (imine), C=O (coumarin), and C=O (hydrazone) groups, respectively, thus confirming the formation of hydrazone coumarin. In addition, the exhibited typical signals in the aromatic region associated with H_4_ at *δ*H 8.15 (s, 1H), H_5_ at *δ*H 7.57 (d, *J* = 8.4 Hz, 1H), H_6_ at *δ*H 6.83 (d, *J* = 8.4 Hz, 1H), and H_8_ at *δ*H 6.76 (s, 1H) of a coumarin moieties. All the protons correlated with carbon signals at *δ*C 142.7, 131.1, 114.1, and 102.3 ppm in the HSQC spectrum (Figure 4, Pact 1), respectively. Finally, the HMBC (Figure 4, Pact 2) from correlations of NH (*δ*H10.78) to C_3__′_ (*δ*C154.4) and H_4_ (*δ*H 8.15) to C_3__′_ (*δ*C154.4) confirmed the expected final structure 7-hyd.HC.

### 2.6. Animals

Thirty-two 10- to 12-week-old male Wistar rats (270 ± 10 g) were used to study the cardioprotective activity of 7-hyd.HC. The animals were purchased from the Central Pharmacy of Tunisia and kept in cages under standard condition (temperature: 20 ± 2°C; humidity: 60 ± 5%; 12 h dark/light cycle) for one-week acclimatization period. The rats were fed with standard chow diet (Table 2) with free access to food and water (*ad libitum*) for 1 week before and during the experiments. The Animal Ethics Committee, University Gafsa, has approved the animal study for this project.

### 2.7. Preparation of Isoproterenol Dose Treatments

The powder of isoproterenol was freshly prepared in distilled water at the time of induction of MI. Isoproterenol suspension (85 mg/kg) was injected via a subcutaneously (s.c) route in rats at 6th and 7th days with an interval of 24 h to induce MI [21].

### 2.8. Rat Groupings and Treatments

The animals were divided into 4 groups (*n* = 8). The pretreated normal control (Control) group received orally 1 mL of NaCl (0.9%) daily, during 7 days. The induced group (ISO) was pretreated orally with saline water and subcutaneously injected with isoproterenol (85 mg/Kg bw) for two consecutive days (6th and 7th days). The positive control group (ISO+Sin) was given sintrom at the dose of 150 *μ*g/Kg bw by gastric gavages, respectively, for 7 days, and in the 6th day and 24 hours later, they were subcutaneously injected with isoproterenol (85 mg/Kg bw). The last group (ISO+7-hyd.HC) was pretreated with the synthesized coumarin 7-hyd.HC at the dose of 150 *μ*g/Kg bw by gastric gavages, respectively, for 7 days, and in the 6th day and 24 hours later, they were injected with isoproterenol (85 mg/Kg bw. s.c) [21]. All rats were euthanized after 48 hours after isoproterenol-induced cardiotoxicity.

### 2.9. Acute Toxicity Study

The control group received distilled water orally while the other groups received different doses of 7-hyd.HC (10, 50, 100, and 150 *μ*g/kg bw) and observed for toxic symptoms and death rate within 12 and 24 h.

### 2.10. Electrocardiography

At the end of the experimental period, needle electrodes were inserted under the skin of the rats in lead II position after anesthesia with ketamine hydrochloride (100 mg/kg body weight) [20]. ECG pattern was made using veterinary electrocardiograph (BIOPAC, Santa Barbara, California), and changes in ECG recordings were considered.

### 2.11. Evaluation of Heart Weight Index

At the end of ECG recordings, the rats were sacrificed, and the heart tissues were excised, washed with NaCl solution, and weighed after blotting with filter paper. The heart weight index (HWI) was calculated as HWI = heart weight (HW)/body weight (BW).

### 2.12. Biochemical Determinations

The rats of each group were scarified; the blood was collected and centrifuged at 2000 g for 15 minutes to separate the plasma. The obtained plasma was kept at 4°C for analysis of several biochemical parameters, including creatine phosphokinase-MB (CK-MB), lactate dehydrogenase (LDH), alanine aminotransferase (ALT), and aspartate amino-transferase (AST). All the analyses were performed using Hitachi 902 Automatic Analyzer using the adapted reagents from Biolabo, France, at the clinic pathological laboratory of the Hospital of Gafsa. The levels of total cholesterol (TC), triglycerides (TG), low-density lipoprotein cholesterol (LDL-C), and high-density lipoprotein cholesterol (HDL-C) were determined using the corresponding commercial kits (Biolabo Reagents, Maizy, France) on an automatic biochemistry analyzer (Kenza, Maizy, France). The concentration of plasma cardiac troponin T was measured using a standard kit by electrochemiluminescence immunoassay (Roche Diagnostics GmbH, Mannheim, Germany). The plasma fibrinogen amount was measured using spectrophotometry-based methods and according to the manufacturer's instructions of the commercial reagent kits purchased from Biomaghreb (Tunisia).

### 2.13. Estimation of Oxidative Stress Markers in the Heart Tissues

The heart tissues of the rats were harvested on the ice, washed with normal saline, and homogenized in aqueous potassium buffer (0.1 M, pH 7.4). The mixture was centrifuged at 12,000 rpm (4°C) for 15 min, and the supernatant was recuperated. The lipid peroxidation was evaluated by the quantification of thiobarbituric acid-reactive substances (TBARS) using the method described by Buege and Aust [22]. Enzymatic antioxidant (SOD, CAT, and GPx) activities were investigated by the methods of Marklund and Marklund [23], Aebi [24], and Flohe and Gunzler [25], respectively. The nonenzymatic antioxidants such as GSH and the protein contents in the heart tissue homogenate were performed by the method of Ellman [26] and Bradford [27], respectively.

### 2.14. Myocardial Expression of Proapoptotic and Antiapoptotic Genes by RT-PCR Analysis

The RT-PCR analysis was performed to validate the differential expression of myocardial genes (caspases-3, B-cell lymphoma 2 (Bcl-2), and B-cell lymphoma-2-associated x (Bax)) as previously described by Prince and Hemalatha [28]. The total RNA sample from the heart tissue of control and experimental rats was extracted by an Easy spin column kit (170-8898, Bio-Rad) according to the manufacturer's protocol. Reverse transcription was performed by 2 *μ*g of the total RNA using superscript reverse transcriptase (Invitrogen, France). Amplification was carried out by the Medox PCR Master Mix in a volume of 25 *μ*l. The real-time cycler conditions were as follows: first denatured at 95°C for 5 min and then amplified with 26 cycles (each cycle was denatured at 94°C for 2 min followed by annealing at 55°C and extension at 72°C for 1 min). The rat sense and antissense primers used in RT-PCR are mentioned in Table 3. Glyceraldehyde-3-phosphate dehydrogenase (GAPDH) was used as a control for this protocol. The RT-PCR products were separated by electrophoresis in 3% agarose gel and visualized by staining with ethidium bromide. The amplicons were quantified by Image J (NIH, MD, USA). Gene expression was quantified relative to the values of the control group after adjusting for GAPDH.

### 2.15. Measurement of Myocardial Infarction Size

The myocardial infarct size was determined as previously described by al-Taweel et al. [29]. Briefly, samples of the heart tissue were incubated in 1% 2,3,5-triphenyltetrazolium chloride (TTC) dissolved in PBS at 37°C for 20 min. The viable heart tissue sections appeared red, while the ischemic region appeared white.

### 2.16. Histopathological Observations

The heart samples of the rats were fixed in 10% buffered formalin. After fixation, the cardiac tissues were dehydrated in a graded series of alcohol, cleared in xylene, and embedded in paraffin. Multiple 5 *μ*m sections from each block were mounted on slides, then stained with hematoxylin and eosin (H&E). The sections were examined under a light microscope and then photographed for histopathological changes.

### 2.17. Statistical Analysis

Results were expressed as mean ± standard deviation (mean ± SD). All analyses were carried out with GraphPad Prism 4.02 for Windows (GraphPad Software, San Diego, CA). Significant differences between treatment effects were determined by one-way analysis of variance (ANOVA), followed by Tukey's test to correct for multiple comparisons with an acceptable statistical level of significance set to 0.05.

## 3. Results

### 3.1. Acute Toxicity

Animals did not show any clinical signs of toxicity up to a dose of 150 *μ*g/kg bw. At this selected dose of 7-hyd.HC, all animals survived and no mortality was observed until the end of the experiment.

### 3.2. Evaluation of Body Weight and Relative Heart Weight

Table 4 shows that the average body weights of the experimental rats were not affected by isoproterenol, sintrom, and 7-hyd.HC. Isoproterenol significantly increased the heart weight/body weight ratio by 19.29% compared to the negative control group. Precotreatment with 7-hyd.HC or sintrom at dose of 150 *μ*g/kg bw significantly reduced the increased relative heart weight by 7.51% and 14.6%, respectively, as compared to untreated MI rats.

### 3.3. Impact of 7-hyd.HC on ECG Pattern

**Figure 5:**
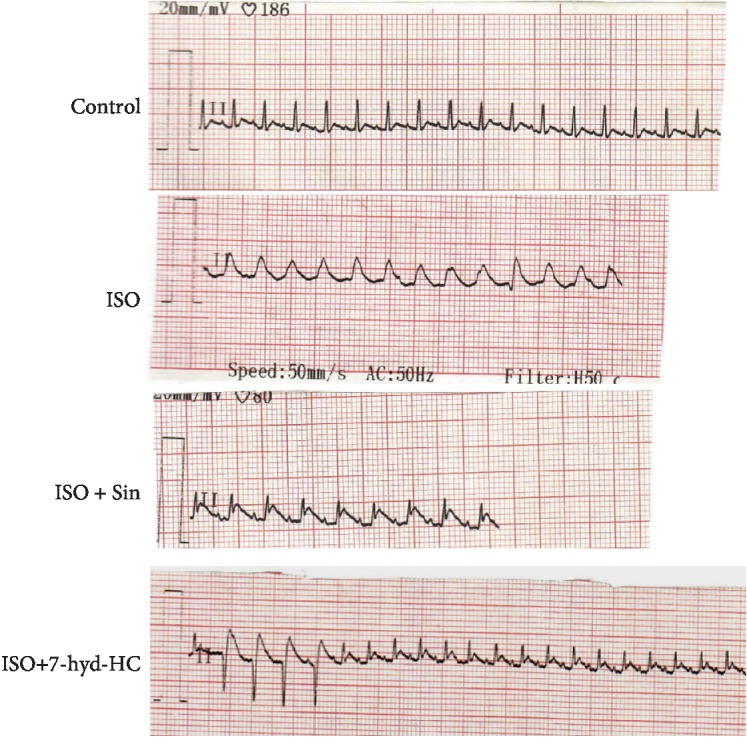
Impact of 7-hyd.HC precotreatment on the electrocardiogram (ECG) patterns of control and experimental groups of rats. The ECG pattern of the negative control group revealed normal electrocardiogram. The ECG pattern of the untreated MI group (ISO) showing pathological changes including a ST-segment elevation and decrease in the R wave amplitude. The ECG pattern of the positive reference group of rats (ISO+Sin) showing a discrete ST-elevation (Pardee wave). The ECG pattern of the ISO+7-hyd.HC group of rats revealed an almost normal sinusal rhythm of the heart.

The ECG pattern of normal and experimental rats is shown in Figure 5. As the ECG profile from the same group is totally similar, we have selected from each group one ECG. Control animals revealed a normal electrocardiographic pattern as evidence by a regular sinus rhythm and normal heart rate (375 ± 13.92 bpm). The ECG of infarcted animals exhibited irregular rhythm with increase in heart rate (430 ± 11.65 bpm), significant elevation of ST-segment and unidentifiable P wave as compared to the negative control group. Oral precotreatment of isoproterenol-induced rats with 7-hyd.HC or sintrom at dose of 150 *μ*g/kg bw exhibited slow-down in heart rate (370 ± 12.34 bpm), decrease in ST segment elevation with identifiable P wave as compared to untreated MI rats.

### 3.4. Impact of 7-hyd.HC on Plasma Cardiac Biomarkers

The effect of 7-hyd.HC on heart marker enzymes (CK-MB, LDH, AST, ALT and troponin-T) is displayed in Table 5. The data revealed that the negative control group demonstrated a normal range of theses biomarkers levels. Isoproterenol injection increased the plasma CK-MB, ALT, AST, LDH and troponin-T by 28%, 32%, 25%, 29% and 31%, respectively, as compared to normal animals. However, precotreatment with 7-hyd.HC followed by isoproterenol-induced MI in rats significantly decreased the amount of plasma CK-MB, ALT, AST, LDH and troponin-T by 12%, 12%, 7%, 11% and 40%, respectively, as compared to infarcted rats.

### 3.5. Impact of 7-hyd.HC on Fibrinogen Level

**Figure 6:**
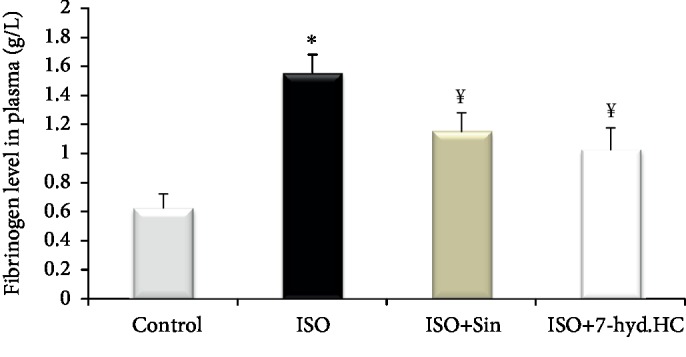
Impact of 7-hyd.HC precotreatment on the fibrinogen level in the plasma of control and experimental groups of rats. Values are expressed as mean ± SD of eight rats in each group. ^∗^*p* < 0.05 significant differences compared to controls; ^¥^*p* < 0.05 significant differences compared to the ISO group of rats.

The levels of plasmatic fibrinogen in the normal and treated groups are shown in Figure 6. ISO-treated rats showed significant (*p* < 0.05) elevated amount of fibrinogen in plasma by 128% compared to the normal group. Precotreatment with sintrom in infracted rats revealed marked (*p* < 0.05) decrease in the levels of plasmatic fibrinogen by 26% as compared to untreated MI rats. Moreover, prior administration of synthesized hydrazone coumarin followed by ISO-induced MI in rats was more effective in reducing the level fibrinogen (by 34%) as compared to the ISO+Sin-treated group.

### 3.6. Lipid Profile Analysis

**Figure 7:**
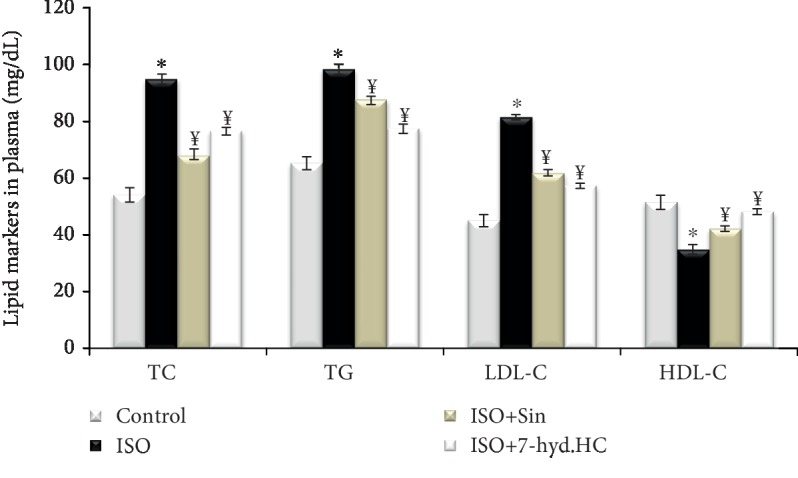
Impact of 7-hyd.HC precotreatment on lipid markers (total cholesterol (TC), triglycerides (TG), low-density lipoprotein cholesterol (LDL-C), and high-density lipoprotein cholesterol (HDL-C) in the plasma of control and experimental groups of rats. Values are expressed as mean ± SD of eight rats in each group. ^∗^*p* < 0.05 significant differences compared to controls; ^¥^*p* < 0.05 significant differences compared to the ISO group of rats.

The lipid profiles obtained are shown in Figure 7. It was observed that the isoproterenol induced significant elevation of the total cholesterol (TC), triglycerides (TG) and LDL-cholesterol (LDL-C) levels by 76%, 51% and 81%, respectively, and significant diminution of HDL-cholesterol (HDL-C) level by 32%, compared with the normal group. Rats precotreated with 7-hyd.HC showed marked decrease of TC, TG and LDL-C by 19%, 21% and 30%, respectively, with an increase of HDL-C by 38% as compared to isoproterenol-induced infarcted rats. Similar, the administration of sintrom significantly restored the levels of these plasmatic lipid profiles as compared to the isoproterenol group.

### 3.7. Effect of 7-hyd.HC on Oxidative Stress Markers in ISO-Induced MI in Rats

The levels of oxidative stress indicator (TBARS) and the activities of SOD, CAT, GSH-Px, and GSH in the heart of the different experimental groups are shown in Table 6. The ISO-treated group revealed significant increase in myocardial oxidative stress markers (TBARS) by 138% compared to the negative control group (*p* < 0.05). Precotreatment either with 7-hyd.HC or sintrom in isoproterenol infarcted rats significantly decreased the TBARS content by 40% and 30%, respectively, compared to the ISO-treated group. Concerning endogenous antioxidants, ISO-treated rats significantly reduced myocardial GSH contents by 34%, as well as SOD, GSH-Px, and catalase activities by 41%, 58%, and 49%, respectively, compared to the negative control group. On the contrary, precotreatment either with 7-hyd.HC or sintrom normalized myocardial GSH levels, and SOD, GSH-Px and catalase activities, compared to untreated MI rats.

### 3.8. Assessment of 7-hyd.HC Effect on Caspase-3, Bcl-2, and Bax Expressions

**Figure 8:**
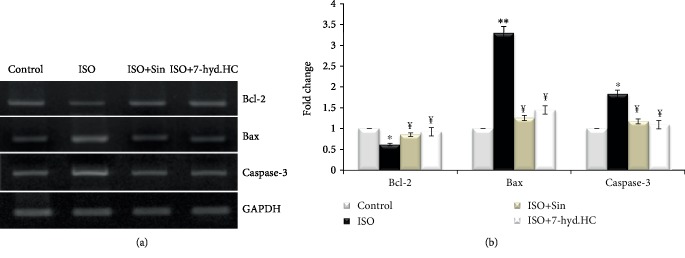
Myocardial expression of proapoptotic and antiapoptotic genes (Caspase-3, Bax, and Bcl2) by RT-PCR (a) and quantification of these genes (b). Values are expressed as mean ± SD of eight rats in each group. ^∗^*p* < 0.05; ^∗∗^*p* < 0.01 significant differences compared to controls; ^¥^*p* < 0.05 significant differences compared to the ISO group of rats.

The impact of 7-hyd.HC on the expression of Bax, Bcl-2, and Caspase-3 in the myocardial tissues of normal and experimental rats by RT-PCR are shown in Figure 8(a). Densitometric analysis of these pro-apoptotic and antiapoptotic markers is shown in Figure 8(b). It was observed that the myocardial expression of Caspase-3 and Bax was significantly enhanced and the expression of Bcl-2 decreased in the isoproterenol-induced myocardial infarcted rats (ISO), when compared to the myocardium of control rats. Oral precotreatment of isoproterenol-induced rats with 7-hyd.HC (ISO+7-hyd.HC) or sintrom (ISO+Sin) downregulated the expression of Caspase-3 and Bax genes and upregulated the expression of Bcl-2 gene as compared to untreated MI rats.

### 3.9. Determination of Myocardial Infarction Size

**Figure 9:**
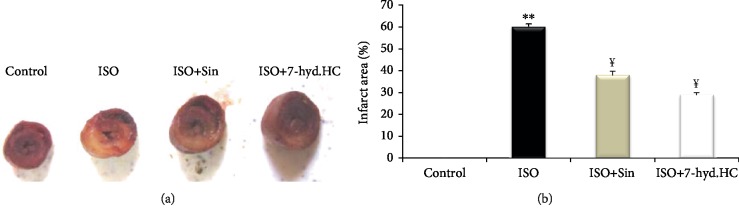
Photomicrographs of myocardial tissue in control and experimental treated rats in TTC staining (a) and scored (b) by the semiquantitative percentage of the myocardial infarct size. Values are expressed as mean ± SD of eight rats in each group. ^∗∗^*p* < 0.01 significant differences compared to controls; ^¥^*p* < 0.05 significant differences compared to the ISO group of rats.

The analyses of myocardial infarction size in control and experimental rats by TTC method are shown in Figure 9(a). The myocardial tissue of control rats appeared normal and red. However, in the isoproterenol-induced myocardial infarcted rats (ISO), a large white region (necrotic patches) was revealed, as compared to the control group. Precotreatment either with 7-hyd.HC or sintrom revealed only less necrotic patches, compared with untreated MI rats.  Figure 9(b) shows the enhanced infarction area in isoproterenol infarcted rats, which was significant (*p* < 0.001) compared with the control group. Rats precotreated with 7-hyd.HC or sintrom showed a marked decrease (*p* < 0.01) in the infarction area as compared to isoproterenol-induced infarcted rats.

### 3.10. Effect of 7-hyd.HC Precotreatment on Histological Changes in ISO-Induced MI in Rats

**Figure 10:**
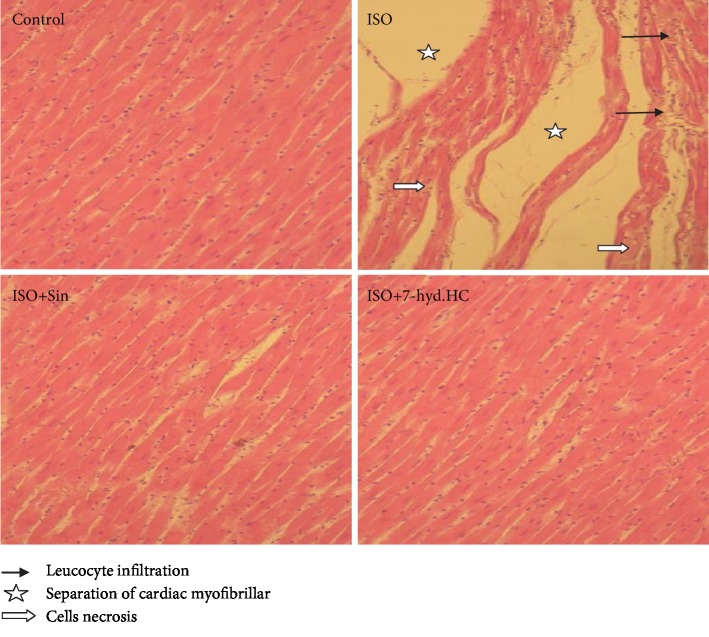
Impact of 7-hyd.HC precotreatment on histology of myocardial tissue (H&E X 400). The control group revealed normal myocardial structure with clear transverse striations. The ISO-treated group showing myocardial cell necrosis with separation of cardiac myofibrillar and excessive leukocyte infiltration. The ISO+Sin-treated group showing moderate injury with few inflammatory cell infiltration. ISO+7-hyd.HC showing normal myocardial arrangement and limited focal neutrophil infiltration in a small area.

The myocardial tissues of the all treated rats were histopathologically examined using hematoxylin-eosin staining (Figure 10). Analysis of heart sections from control treated rats showed normal myocardium architecture and regular cell distribution. While, as compared to the normal group, the histological examination of the heart from ISO-intoxicated rats showed a significant loss of myofibrils, extensive inflammation, areas of edema, degenerated vacuolated myocytes, and leucocyte infiltration, on the contrary, precotreatment either with 7-hyd.HC or sintrom revealed only few occasional inflammatory cells and focal vacuolization in the myocytes, which demonstrated the protective efficiency of 7-hyd.HC or sintrom against the cardiotoxic effect of ISO.

## 4. Discussion

Oxidative stress has been considered a conjoint pathological mechanism, and it contributes to initiation and progression of various cardiovascular dysfunctions, such as myocardial infarction (MI) [30, 31]. Moreover, many researchers have suggested that oxidative stress induces amplification of the inflammatory response and apoptosis leading to the initiation of ischemic lesion [32, 33]. Thus, the use of antioxidant drugs as a scavenger of free radicals may reduce the extent of myocardial damage [34]. Recently, several coumarins based natural and synthetic derivatives have been used as anti-inflammatory, anticoagulant, antithrombotic, antioxidant, and antihyperlipidemic [14, 35, 36]. Hence, our study investigated the effects of precotreatment with (E)-N′-(1-(7-hydroxy-2-oxo-2H-chromen-3-yl) ethylidene) benzohydrazide, a potent synthesized hydrazone coumarin, on the changes in oxidative parameters and cardiac biomarkers levels, myocardial proapoptotic and antiapoptotic factors, and the modification on histopathological parameters and heart function, during ISO-induced MI in rats.

Results clearly showed that the body weight of the rats was not affected by the isoproterenol treatment. The obtained data were in line with the findings reported by Mnafgui et al. [37] who suggested that isoproterenol did not induce changes in the body weight of rats in the acute MI study. Data showed that the increase in the relative weight of the heart of the isoproterenol-induced myocardial infarction group was significantly improved by 7-hyd.HC precotreatment. The observed cardiac hypertrophy could be due to an invasion of inflammatory cells associated with fibrose [5, 20], which was confirmed by biochemical and histological evaluations.

Furthermore, the ECG pattern in ISO-treated groups revealed ischemic and conduction abnormalities as evidenced by significant ST elevation together with unidentifiable P wave. The observed conduction abnormalities might be associated with necrosis of cardiac muscle fibers [4, 38]. Additionally, the remarkable elevation in the heart rate is supposed to cause the increase of oxygen consumption which then leads to accelerated myocardial necrosis [39]. Results showed also that the precotreatment with 7-hyd.HC restored normal ECG recordings. It seemed that the 7-hyd.HC neutralized the effect of isoproterenol on the myocardium tissue and coronary vessels, as has been suggested previously by Ghazouani et al. [21] who reported the beneficial effect of a synthesized coumarin towards the cell membrane in infarcted rats.

The toxic effect of isoproterenol together with the beneficial effect of the 7-hyd.HC was confirmed by a biochemical and a histological examinations in the present work. The current findings revealed that isoproterenol treatment induced deterioration in the cardiac function, revealed by an increased in the plasma levels of troponin-T, LDH, ALT, AST, and CK-MB activities, indicating a severe damage of the myocardium cell and leakiness of the plasma membrane. These findings were in accordance with previous studies [40, 41]. It was interesting that 7-hyd.HC precotreatment revealed a significant decrease in the amounts of all these cardiac markers, which was also observed in the Sin-treated group. The current findings indicated a cardiopreventive role of the 7-hyd.HC by the maintenance of the myocardium membrane integrity and therefore restricting the leakage of these enzymes into the bloodstream [21].

Oxidative stress is recognized as the major inducer in the progression of MI [42]. In the present work, ISO-treated groups showed a significant increase in the oxidative stress markers (TBARS) with subsequent depletion of endogenous antioxidants (GSH, SOD, CAT, and GPx), which is known as a experimental and clinical marker of tissue damage [43, 44]. The appearance of cardiac oxidative stress might be due to free radical production which mediate myocardial membrane dysfunction following isoproterenol intoxication [28]. However, precotreatment with 7-hyd.HC lessened the oxidative stress produced by ISO, which reduced lipid peroxidation, and increased the activities of the studied antioxidant enzymes (SOD, CAT, and GPx), as well as GSH contents. Therefore, the inhibition of lipid peroxidation with activation of antioxidant activities is considered a protection from MI [2]. The beneficial role of 7-hyd.HC seemed to be due to the scavenging of reactive oxygen species generated by the metabolism of isoproterenol, which could protect the cardiac tissue from oxidative stress-induced injury. In fact, 7-hyd.HC exerts their strong antioxidant capacity owing to their design and construction, thereby known for its radical scavenging activities [20, 21].

Literature suggested that cardiomyocyte apoptosis induced by oxidative stress plays a significant role in the cardiac tissue damage and progression of myocardial infarction [6]. The protein like Bcl-2 is a key regulatory component which protect cells from apoptosis, while Bax and caspase-3, as proapoptotic genes, promotes cell death [29]. Indeed, oxidative stress activates pathways of apoptosis through upregulating Bax protein and caspase enzyme and downregulating the antiapoptotic Bcl-2 [28]. This hypothesis was strengthened by the present study showing that ISO treatment significantly increased the expression of Bax and caspase-3 genes and reduced the expression of Bcl-2 protein compared to the control group, a phenomenon which could increase apoptosis and result in functional abnormalities of the myocardium.

The inhibition of caspase and Bax activation is one of the major approaches to attenuate myocardial apoptosis [45]. 7-hyd.HC precotreatment inhibited oxidative stress, by its antioxidant effect, and increased the expression of Bcl-2 genes in the myocardium and decreased the expression of Bax and caspase-3 genes in ISO-induced myocardial infarcted rats, thereby protected the myocardial cells from apoptosis.

In addition, the accumulation of free radicals in the heart after an MI can cause cardiac tissue damage to multiple degrees and lead to cell death [46]. Accordingly, histopathological examination of the MI group evidenced focal ischemic lesions in the myocardium and interstitial edema and cellular necrosis which were in accordance with previous data [39]. The 7-hyd.HC by its proven antioxidant capacity may have ameliorative effects on the abolition of myocardium cell necrosis, which could be attributed to their antioxidant potential [19]. Indeed, the structure of this new synthesized hydrazone coumarin containing different privileged substructures within the same molecule (coumarin) and function (hydrazone) represents an important strategy to fight against oxidative stress [20].

In the present study, a significant perturbation of the lipid profile was observed in rats receiving supramaximal doses of isoproterenol, as observed by a remarkable rise in plasma TC, TG, and LDL-C and considerable decrease in HDL-C. These changes in lipid parameters are considered as a risk of developing ischemic heart disease [47]. However, a strong inhibition of the alteration of lipid profile by 7-hyd.HC was observed in the plasma of ISO+7-hyd.HC-treated rats. Therefore, the hypolipidemic capacity of 7-hyd.HC may be due to the inhibitory effects on pancreatic lipase, the most important enzyme in digestion of fat [37, 48]. In fact, the action of 7-hyd.HC in lowering lipid could be a strategy to prevent and treat MI [10]. Plasma fibrinogen is a major determinant of platelet aggregation and blood viscosity. The increase in plasma fibrinogen concentrations is associated with an increase in the risk of cardiovascular diseases and myocardial infarction [49, 50]. The current data were in agreement with those of previous findings [13]. The isoproterenol-treated group exhibited a remarkable elevated amount of plasma fibrinogen as compared to the negative control group, which explained the necrosis and ischemic development observed in the myocardium tissue of the infarcted rats [51]. Precotreatement with 7-hyd.HC effectively restored the normal level of fibrinogen in plasma of infarcted rats. A similar effect has been observed by Ghazouani et al. [21]. The antithrombotic effect of 7-hyd.HC may be attributed to its coumarin nature by acting as a vitamin K antagonist (VKA) [20]. In fact, the mechanism underlying the potential antiembolic effect exerted by 7-hyd.HC is a result of the inhibition of the vitamin K-dependent gamma-carboxylation of diver coagulation factors (II, VII, IX, and X), resulting in the formation of biologically inactive forms of these coagulation proteins, as has been reported previously [52, 53, 54].

## 5. Conclusion

The present study was conducted in order to find a new coumarin derivative against the cardiovascular trouble. Results showed that the tested drug 7-hyd.HC exerted a significant cardiopreventive effect against isoproterenol-induced MI, as demonstrated by ECG, biochemical, molecular, and histological examinations. It was clear that 7-hyd.HC reduced the lipid peroxidation effects and ameliorated the myocardial endogenous antioxidant activities. This antioxidative capacity of 7-hyd.HC led to the improvement of the cardiac biomarkers and the decrease of the level of fibrinogen which induced a reduction in the inflammatory pathways. In addition, 7-hyd.HC prevented cardiomyocyte apoptosis by modulating Bcl-2, Bax, and caspase-3 gene expressions. Furthermore, 7-hyd.HC exerted better antioxidative effects on MI compared to conventional sintrom, which can lead to improved myocardial function and attenuated the ischemic lesions of myocardium tissue. These results highlighted new insights into the development of a novel therapeutic target for cardiovascular diseases.

## Figures and Tables

**Table 1:** 1D and 2D NMR data of compound 7-hyd.HC in DMSO-d6.

Position	*δ*H ppm (mult, J(Hz))	*δ*C ppm	HMBC correlations
2	—	160.1	H4 (8.15)
3	—	122.4	CH3 (2.32); H4 (8.15)
3'	—	154.4	CH3 (2.32); H4 (8.15); NH(10.78)
4	8.15 (s, 1H, H4)	142.7	H5 (7.57)
4a	—	111.6	H4 (8.15); H5 (7.57); H6 (6.83); H8 (6.76)
5	7.57 (d, *J* = 8.4 Hz, 1H, H5)	131.1	H4 (8.15); H6 (6.83)
6	6.83 (d, *J* = 8.4 Hz, 1H, H6)	114.1	H5 (7.57); H8 (6.76)
7	10.78 (s, 1H, OH)	162.4	H5 (7.57); H6 (6.83); H8 (6.76)
8	6.76 (s, 1H, H8)	102.3	H6 (6.83)
8a	—	156.0	H4 (8.15); H5 (7.57); H8 (6.76)
9	—	164.5	H11 (7.88); NH(10.78)
10	—	134.4	H11 (7.88); H12(7.51)
11	7.88 (d, *J* = 6.8 Hz, 2H, H11)	128.4	H12 (7.51); H13 (7.68)
12	7.51 (t, *J* = 6.8 Hz, 2H, H12)	128.7	H11 (7.88); H13 (7.68)
13	7.68 (t, *J* = 6.8 Hz, 1H, H13)	132.0	H11 (7.65); H12 (7.51)
CH_3_	2.32 (s, 3H, CH_3_)	16.8	—
NH	10.78 (s, 1H,NH)	—	—

**Table 2:** Energy resources (kJ/g) and ratio (%) of the diets.

	Protein	Carbohydrate	Fat	Total
Ratio (%) of normal chow diet	21.6	65.6	12.8	—
Energy resources (kJ/g)	3.31	10.04	1.95	15.3

**Table 3:** Primers used in RT-PCR study.

Gene	Primers
Caspase-3	Sense primer: CAG AGC TGG ACT GCG GTA TTG A Antisense primer: AGC ATG GCG CAA AGT GAC TG

Bax	Sense primer: TTC ATC CAG GAT CGA GCA GA Antisense primer: GCA AAG TAG AAG GCA ACG

Bcl-2	Sense primer: CTG GTG GAC AAC ATC GCT CTG Antisense primer: GGT CTG CTG ACC TCA CTT GTG

**Table 4:** Energy intakes and heart weight index (HWI) in control and experimental rats.

	Control	ISO	ISO + sin	ISO+ 7-hyd.HC
Daily energy intakes (kJ/day/rat)	344.6 ± 1.38	341.9 ± 1.6	343.4 ± 2.3	342.2 ± 0.81
Body weight (g)	310.3 ± 1.9	313.3 ± 2.13	312.7 ± 3.28	314.2 ± 1.92
Heart weight (g)	1.37 ± 0.01	1.65 ± 0.02^∗^	1.52 ± 0.06^¥^	1.41 ± 0.01^¥^
Heart weight index (HWI)	0.44 ± 0.01	0.52 ± 0.01^∗^	0.48 ± 0.01^¥^	0.44 ± 0.02^¥^

Values are expressed as mean ± SD of eight rats in each group. ^∗^*p* < 0.05 significant differences compared to controls; ^¥^*p* < 0.05 significant differences compared to the ISO group of rats.

**Table 5:** Impact of 7-hyd.HC precotreatment on the creatine kinase-MB (CK-MB), lactate dehydrogenase (LDH), aspartate aminotransferase (AST), alanine aminotransferase (ALT), and troponin-T (Tn-T) levels in the plasma of control and experimental groups of rats.

Parameters	Control	ISO	ISO+Sin	ISO+7-hyd.HC
CK-MB (U/L)	182.7 ± 2.76	235.0 ± 2.51	229.1 ± 1.53^∗^	205.7 ± 2.67^¥^
LDH (U/L)	160.9 ± 1.18	212.0 ± 1.31	195.4 ± 0.80^∗^	188.5 ± 2.14^¥^
AST (U/L)	137.0 ± 1.17	196.9 ± 1.39	188.5 ± 1.30^∗^	184.3 ± 1.4^¥^
ALT (U/L)	83.05 ± 1.50	104.4 ± 1.64	86.78 ± 1.62^∗^	91.52 ± 1.42^¥^
Tn-T (ng/mL)	0.15 ± 0.1	1.6 ± 0.21	1.22 ± 0.22^∗^	0.96 ± 0.23^¥^

Values are expressed as mean ± SD of eight rats in each group. ^∗^*p* < 0.05 significant differences compared to controls; ^¥^*p* < 0.05 significant differences compared to the ISO group of rats.

**Table 6:** Impact of 7-hyd.HC precotreatment on lipid peroxidation products and endogenous antioxidants in the heart tissues of the control and experimental groups of rats.

Parameters	Control	ISO	ISO+Sin	ISO+7-hyd.HC
TBARS (nmoles MDA/g tissue)	6.87 ± 0.56	16.34 ± 0.63	9.81 ± 0.44^∗^	11.49 ± 0.51^¥^
GSH (*μ*moles/g tissue)	8.87 ± 0.46	5.78 ± 0.43	6.97 ± 0.24^∗^	7.43 ± 0.27^¥^
CAT (*μ*mol of H_2_O_2_ destroyed/min per mg protein)	4.12 ± 0.54	2.12 ± 0.23	3.01 ± 0.12^∗^	3.74 ± 0.09^¥^
SOD (U/mg protein)	6.53 ± 0.45	3.85 ± 0.33	5.92 ± 0.1^∗^	6.15 ± 0.18^¥^
GPx (nmol of NADPH oxidized/min per mg protein)	4.75 ± 0.17	1.99 ± 0.07	2.94 ± 0.1^∗^	3.52 ± 0.09^¥^

Values are expressed as mean ± SD of eight rats in each group. ^∗^*p* < 0.05 significant differences compared to controls; ^¥^*p* < 0.05 significant differences compared to the ISO group of rats.

## Data Availability

All the data supporting the results are shown in the paper and can be available from the corresponding author.

## References

[1] World Health Organization (WHO) (2015). *Fact Sheet No. 317*.

[2] Shaikh S., Bhatt L. K., Barve K. (2019). Attenuation of isoproterenol-induced cardiotoxicity in rats by Narirutin rich fraction from grape fruit. *Phytomedicine*.

[3] Song S., Si L. (2015). Klotho ameliorated isoproterenol-induced pathological changes in cardiomyocytes via the regulation of oxidative stress. *Life Sciences*.

[4] Attalla D. M., Ahmed L. A., Zaki H. F., Khattab M. M. (2018). Paradoxical effects of atorvastatin in isoproterenol-induced cardiotoxicity in rats: role of oxidative stress and inflammation. *Biomedicine & Pharmacotherapy*.

[5] Prasad E. M., Mopuri R., Islam M. S., Kodidhela L. D. (2017). Cardioprotective effect of Vitex negundo on isoproterenol-induced myocardial necrosis in wistar rats: a dual approach study. *Biomedicine and Pharmacotherapy*.

[6] Sadeghzadeh J., Vakili A., Bandegi A. R., Sameni H. R., Zahedi Khorasani M., Darabian M. (2017). Lavandula reduces heart injury via attenuating tumor necrosis factor-alpha and oxidative stress in a rat model of infarct-like myocardial injury. *Cell Journal*.

[7] Soner B. C., Şahin A. S. (2014). Cardiovascular effects of resveratrol and atorvastatin treatments in an H2O2-induced stress model. *Experimental and Therapeutic Medicine*.

[8] Heusch G., Rassaf T. (2016). Time to give up on cardioprotection?. *Circulation Research*.

[9] Fernandez G., Spatz E. S., Jablecki C., Phillips P. S. (2011). Statin myopathy: a common dilemma not reflected in clinical trials. *Cleveland Clinic Journal of Medicine*.

[10] Daoud A., Ben Mefteh F., Mnafgui K. (2017). Cardiopreventive effect of ethanolic extract of date palm pollen against isoproterenol induced myocardial infarction in rats through the inhibition of the angiotensin-converting enzyme. *Experimental and Toxicologic Pathology*.

[11] Deng X. Y., Chen J. J., Li H. Y., Ma Z. Q., Ma S. P., Fu Q. (2015). Cardioprotective effects of timosaponin B II from *Anemarrhenae asphodeloides* Bge on isoproterenol-induced myocardial infarction in rats. *Chemico-Biological Interactions*.

[12] Dávila-Fajardo C. L., Díaz-Villamarín X., Antúnez-Rodríguez A. (2019). Pharmacogenetics in the treatment of cardiovascular diseases and its current progres regarding implementation in the clinical routine. *Genes (Basel)*.

[13] Kontogiorgis C., Nicolotti O., Mangiatordi G. F. (2015). Studies on the antiplatelet and antithrombotic profile of anti-inflammatory coumarin derivatives. *Journal of Enzyme Inhibition and Medicinal Chemistry*.

[14] Kirsch G., Abdelwahab A. B., Chaimbault P. (2016). Natural and synthetic coumarins with effects on inflammation. *Molecules*.

[15] Najafi Z., Mahdavi M., Saeedi M. (2019). Novel tacrine-coumarin hybrids linked to 1,2,3-triazole as anti-Alzheimer's compounds: *in vitro* and *in vivo* biological evaluation and docking study. *Bioorganic Chemistry*.

[16] Taha M., Shah S. A. A., Afifi M. (2018). Synthesis, *α*-glucosidase inhibition and molecular docking study of coumarin based derivatives. *Bioorganic Chemistry*.

[17] Kasperkiewicz K., Ponczek M. B., Budzisz E. (2018). A biological, fluorescence and computational examination of synthetic coumarin derivatives with antithrombotic potential. *Pharmacological Reports*.

[18] Pérez-Cruz K., Moncada-Basualto M., Morales-Valenzuela J. (2018). Synthesis and antioxidant study of new polyphenolic hybrid-coumarins. *Arabian Journal of Chemistry*.

[19] Yadav N., Agarwal D., Kumar S., Dixit A. K., Gupta R. D., Awasthi S. K. (2018). *In vitro* antiplasmodial efficacy of synthetic coumarin-triazole analogs. *European Journal of Medicinal Chemistry*.

[20] Mnafgui K., Khdhiri E., Ghazouani L. (2019). Anti-embolic and anti-oxidative effects of a novel (E)-4-amino-N′ -(1-(7-hydroxy-2-oxo-2H-chromen-3-yl) ethylidene) benzohydrazide against isoproterenol and vitamin-K induced ischemic stroke. *Archives of Physiology and Biochemistry*.

[21] Ghazouani L., Khdhiri E., Elmufti A. (2019). Cardioprotective effects of (E)-4-hydroxy-N′ -(1-(3-oxo-3H-benzo[f]chromen-2-yl)ethylidene)benzohydrazide: a newly synthesized coumarin hydrazone against isoproterenol-induced myocardial infarction in a rat model. *Canadian Journal of Physiology and Pharmacology*.

[22] Buege J. A., Aust S. D. (1978). [30] Microsomal lipid peroxidation. *Methods in Enzymology*.

[23] Marklund S., Marklund G. (1974). Involvement of the super-oxide anion radical in the autoxidation of pyrogallol and a convenient assay for superoxide dismutase. *European Journal of Biochemistry*.

[24] Aebi H. (1984). [13] Catalase *in vitro*. *Methods in Enzymology*.

[25] Flohe L., Gunzler W. A. (1984). [12] Assays of glutathione peroxidase. *Methods in Enzymology*.

[26] Ellman G. L. (1959). Tissue sulfhydryl groups. *Archives of Biochemistry and Biophysics*.

[27] Bradford M. M. (1976). A rapid and sensitive method for the quantitation of microgram quantities of protein utilizing the principle of protein-dye binding. *Analytical Biochemistry*.

[28] Stanely Mainzen Prince P., Hemalatha K. L. (2018). A molecular mechanism on the antiapoptotic effects of zingerone in isoproterenol induced myocardial infarcted rats. *European Journal of Pharmacology*.

[29] al-Taweel A. M., Raish M., Perveen S. (2017). *Nepeta deflersiana* attenuates isoproterenol-induced myocardial injuries in rats: possible involvement of oxidative stress, apoptosis, inflammation through nuclear factor (NF)-*κ*B downregulation. *Phytomedicine*.

[30] Garson C., Kelly-Laubscher R., Blackhurst D., Gwanyanya A. (2015). Lack of cardioprotection by single-dose magnesium prophylaxis on isoprenaline-induced myocardial infarction in adult Wistar rats. *Cardiovascular Journal of Africa*.

[31] Suchal K., Malik S., Gamad N. (2016). Kampeferol protects against oxidative stress and apoptotic damage in experimental model of isoproterenol-induced cardiac toxicity in rats. *Phytomedicine*.

[32] Chen H., Xu Y., Wang J., Zhao W., Ruan H. (2015). Baicalin ameliorates isoproterenol-induced acute myocardial infarction through iNOS, inflammation and oxidative stress in rat. *International Journal of Clinical and Experimental Pathology*.

[33] Goyal S. N., Sharma C., Mahajan U. B. (2015). Protective effects of cardamom in isoproterenol-induced myocardial infarction in rats. *International Journal of Molecular Sciences*.

[34] Kurian G. A., Rajagopal R., Vedantham S., Rajesh M. (2016). The role of oxidative stress in myocardial ischemia and reperfusion injury and remodeling: revisited. *Oxidative Medicine and Cellular Longevity*.

[35] Paracatu L. C., Zeraik M. L., de Carvalho Bertozo L. (2016). Synthesis, antioxidant and anti-inflammatory properties of an apocynin-derived dihydrocoumarin. *Medicinal Chemistry*.

[36] Srikrishna D., Godugu C., Dubey P. K. (2018). A review on pharmacological properties of coumarins. *Mini-Reviews in Medicinal Chemistry*.

[37] Mnafgui K., Khlif I., Hajji R. (2015). Preventive effects of oleuropein against cardiac remodeling after myocardial infarction in Wistar rat through inhibiting angiotensin-converting enzyme activity. *Toxicology Mechanisms and Methods*.

[38] Kannan M. M., Quine D. S. (2013). Ellagic acid inhibits cardiac arrhythmias, hypertrophy and hyperlipidaemia during myocardial infarction in rats. *Metabolism*.

[39] Mnafgui K., Hajji R., Derbali F. (2016). Anti-inflammatory, antithrombotic and cardiac remodeling preventive effects of eugenol in isoproterenol-induced myocardial infarction in Wistar rat. *Cardiovascular Toxicology*.

[40] Paul S., Das S., Tanvir E. M. (2017). Protective effects of ethanolic peel and pulp extracts of *Citrus macroptera* fruit against isoproterenol-induced myocardial infarction in rats. *Biomedicine and Pharmacotherapy*.

[41] Khan V., Hassan M. Q., Akhtar M., Najmi A. K. (2018). Renin inhibition by aliskiren protects rats against isoproterenol induced myocardial infarction. *Drug Research*.

[42] Ardjmand A., Shahabbodin M. E., Mazoochi T., Heidari A., Ghavipanjeh G. (2019). Cardioprotective effects of cerebrolysin on the lesion severity and inflammatory factors in a rat model of isoproterenol-induced myocardial injury. *Pharmacological Reports*.

[43] Zhang W., Li Y., Ge Z. (2017). Cardiaprotective effect of crocetin by attenuating apoptosis in isoproterenol induced myocardial infarction rat model. *Biomedicine & Pharmacotherapy*.

[44] Garg S., Malhotra R. K., Khan S. I. (2019). Fisetin attenuates isoproterenol-induced cardiac ischemic injury *in vivo* by suppressing RAGE/NF-*κ*B mediated oxidative stress, apoptosis and inflammation. *Phytomedicine*.

[45] Khan V., Sharma S., Bhandari U., Sharma N., Rishi V., Haque S. E. (2019). Suppression of isoproterenol-induced cardiotoxicity in rats by raspberry ketone via activation of peroxisome proliferator activated receptor-*α*. *European Journal of Pharmacology*.

[46] Mnafgui K., Hajji R., Derbali F. (2016). Protective effect of hydroxytyrosol against cardiac remodeling after isoproterenol-induced myocardial infarction in rat. *Cardiovascular Toxicology*.

[47] Abbas A. M. (2016). Cardioprotective effect of resveratrol analogue isorhapontigenin versus omega-3 fatty acids in isoproterenol-induced myocardial infarction in rats. *Journal of Physiology and Biochemistry*.

[48] Pari L., Rajarajeswari N., Saravanan S., Rathinam A. (2014). Antihyperlipidemic effect of coumarin in experimental type 2 diabetic rats. *Biomedicine & Preventive Nutrition*.

[49] Ahad Eshraghian M. (2009). Fibrinogen as a risk factor for premature myocardial infarction in Iranian patients: a case control study. *Vascular Health and Risk Management*.

[50] Walton B. L., Getz T. M., Bergmeier W., Lin F. C., Uitte de Willige S., Wolberg A. S. (2014). The fibrinogen *γ*A/*γ*′ isoform does not promote acute arterial thrombosis in mice. *Journal of Thrombosis and Haemostasis*.

[51] Davalos D., Akassoglou K. (2012). Fibrinogen as a key regulator of inflammation in disease. *Seminars in Immunopathology*.

[52] Pineo G., Hull R. D. (2003). Coumarin therapy in thrombosis. *Hematology/Oncology Clinics of North America*.

[53] Ufer M. (2005). Comparative pharmacokinetics of vitamin K antagonists: warfarin, phenprocoumon and acenocoumarol. *Clinical Pharmacokinetics*.

[54] Trailokya A., Hiremath J. S., Sawhney J. (2016). Acenocoumarol: a review of anticoagulant efficacy and safety. *Journal of the Association of Physicians of India*.

